# Extrasynaptic NMDA receptor dependent long-term potentiation of hippocampal CA1 pyramidal neurons

**DOI:** 10.1038/s41598-017-03287-7

**Published:** 2017-06-08

**Authors:** Qian Yang, Geng Zhu, Dandan Liu, Jue-Gang Ju, Zhen-Hua Liao, Yi-Xin Xiao, Yue Zhang, Naijian Chao, JieJie Wang, Weidong Li, Jian-Hong Luo, Sheng-Tian Li

**Affiliations:** 1 Key laboratory for the Genetics of Developmental and Neuropsychiatric Disorders (Ministry of Education), Bio-X Institutes, Shanghai Key Laboratory of Psychotic Disorders, Institute of Social Cognitive and Behavioral Sciences, and Brain Science and Technology Research Center, Shanghai Jiao Tong University, Shanghai, China; 2 Department of Neurobiology, Key Laboratory of Medical, Neurobiology (Ministry of Health of China), Collaborative Innovation Center for Brain Science, School of Medicine, Zhejiang University, Hangzhou Zhejiang, 310058 China

## Abstract

In the adult mouse hippocampus, NMDA receptors (NMDARs) of CA1 neurons play an important role in the synaptic plasticity. The location of NMDARs can determine their roles in the induction of long-term potentiation (LTP). However, the extrasynaptic NMDARs (ES-NMDARs) dependent LTP haven’t been reported. Here, through the use of a 5-Hz stimulation and MK-801 (an irreversible antagonist of NMDARs) in the CA1 neurons of adult mice hippocampal slices, synaptic NMDARs were selectively inhibited and NMDAR-mediated excitatory postsynaptic currents were not recovered. We found that a robust LTP was induced by 3-train 100-Hz stimulation when the synaptic NMDARs and extrasynaptic NR2B containing NMDARs were blocked, but not in the any of the following conditions: blocking of all NMDARs (synaptic and extrasynaptic), blocking of the synaptic NMDARs, and blocking of the synaptic NMDARs and extrasynaptic NR2A-containing NMDARs. The results indicate that this LTP is ES-NMDARs dependent, and NR2B-containing ES-NMDARs modulates the threshold of LTP induction.

## Introduction

The role of NMDA receptors (NMDARs) in the induction of long-term potentiation (LTP) in the hippocampus is well established^1–4^. NMDARs are primarily heteromeric assemblies of NR1, NR2 and NR3; in particular, the NR2 subunit determines many of the properties and functions of NMDARs. NR2A and NR2B are two predominant NR2 subunits in the hippocampus, and they have a strong dependence on magnesium ions, which present better associativity of LTP than other NR2 subunits^5^. NMDARs exist both at the synapse and on the extrasynaptic membrane; and are referred to as S-NMDAR and ES-NMDAR depending on their location^6, 7^. NR2A- and NR2B-containing receptors were considered to be exclusively segregated to the synaptic (NR2A) and extrasynaptic (NR2B) compartments, but increasing evidence suggests that NR2A and NR2B can be located synaptically or extrasynaptically^8^. The primary subtype of S-NMDARs switches from NR2B to NR2A subunits during postnatal development^9^.

The different subtypes of NMDARs play varied roles in LTP induction, and many studies have especially focused on the NR2A and NR2B subunits. Direct evidence has demonstrated that NR2A is necessary for LTP introduction; for example, the disruption of NR2A resulted in the reduction of LTP and spatial learning in mice^10^, and the inhibition of NR2A-containing NMDARs by NVP-AAM077 prevented the induction of LTP^11^. However, the role of NR2B in LTP is unclear. It reported that the inhibition of NR2B by Ro25-6981 or ifenprodil had no effect on LTP induction in the adult hippocampal CA1 synapse^11^. Although, transgenic over-expression of NR2B in the mice forebrain has been reported to improve LTP^12^, how NR2B over-expression enhances LTP remains unclear. In the adult rat brain, most NR2B subunits express extrasynaptically^9^, and the LTP procedure mainly activates the S-NMDARs in normal physiological conditions. An increasing number of studies have also reported that the postsynaptic location of NMDARs is critical to synaptic plasticity^13–15^. This makes the roles of NR2A and NR2B subunits in synaptic plasticity more complex, and additional studies are warranted.

In certain pathological situations, such as Huntington disease, ES-NMDARs are over-activated or S-NMDARs are inhibited^16–18^. In these diseases, the attenuation of learning and memory abilities is also usually observed. It has been demonstrated that S-NMDARs play an important role in LTP, but whether the activation or inhibition of ES-NMDARs influences LTP remains unknown. Here, by combining a short train of 5-Hz stimulation and an irreversible use-dependent NMDAR antagonist (MK-801), we succeeded in selectively inhibiting S-NMDARs in mature hippocampal slices and found a new kind of LTP which was induced when S-NMDARs and NR2B-containing ES-NMDARs were inhibited.

## Results

### LTP in CA1 neurons when S-NMDARs and extrasynaptic NR2B containing NMDARs were inhibited

**Figure 1:**
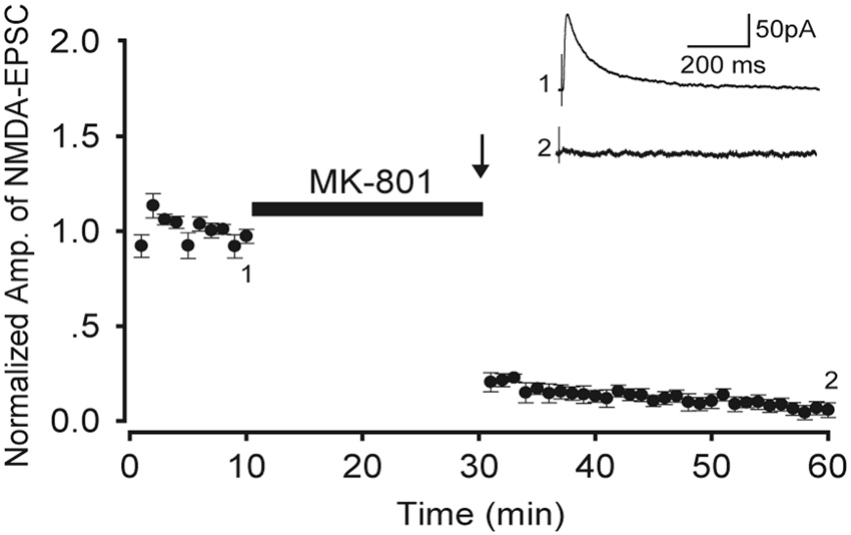
Amplitude of NMDA-EPSCs before and after blocking S-NMDARs. The arrow represents the stimulation time point. Inset: sample traces of NMDA-EPSC at time points 1 and 2, as marked. Data are from nine slices of five mice.

It has been reported that S-NMDARs could be selectively inhibited by the use-dependent NMDARs open channel blocker MK-801 in acute slice preparations^6, 19^. MK-801 binds selectively and with high affinity to NMDARs when they are in their open state^20^, and stimulation less than 10 Hz cannot open ES-NMDARs^21^. In this study, we applied MK-801 for 20 min and then delivered 5-Hz stimulation for 16 s (as shown in Fig. 1), by which that S-NMDARs can be selectively blocked as previously reported^19^. After the MK-801 application and 5-Hz stimulation, the S-NMDARs were blocked and the amplitude of the NMDA-EPSCs recorded through whole-cell patch-clamp was almost zero. Following the washout of MK-801 with normal ACSF, the NMDA-EPSCs were not recovered for at least 30 min in all nine tested slices, indicating that the blockade of S-NMDARs is stable.

**Figure 2:**
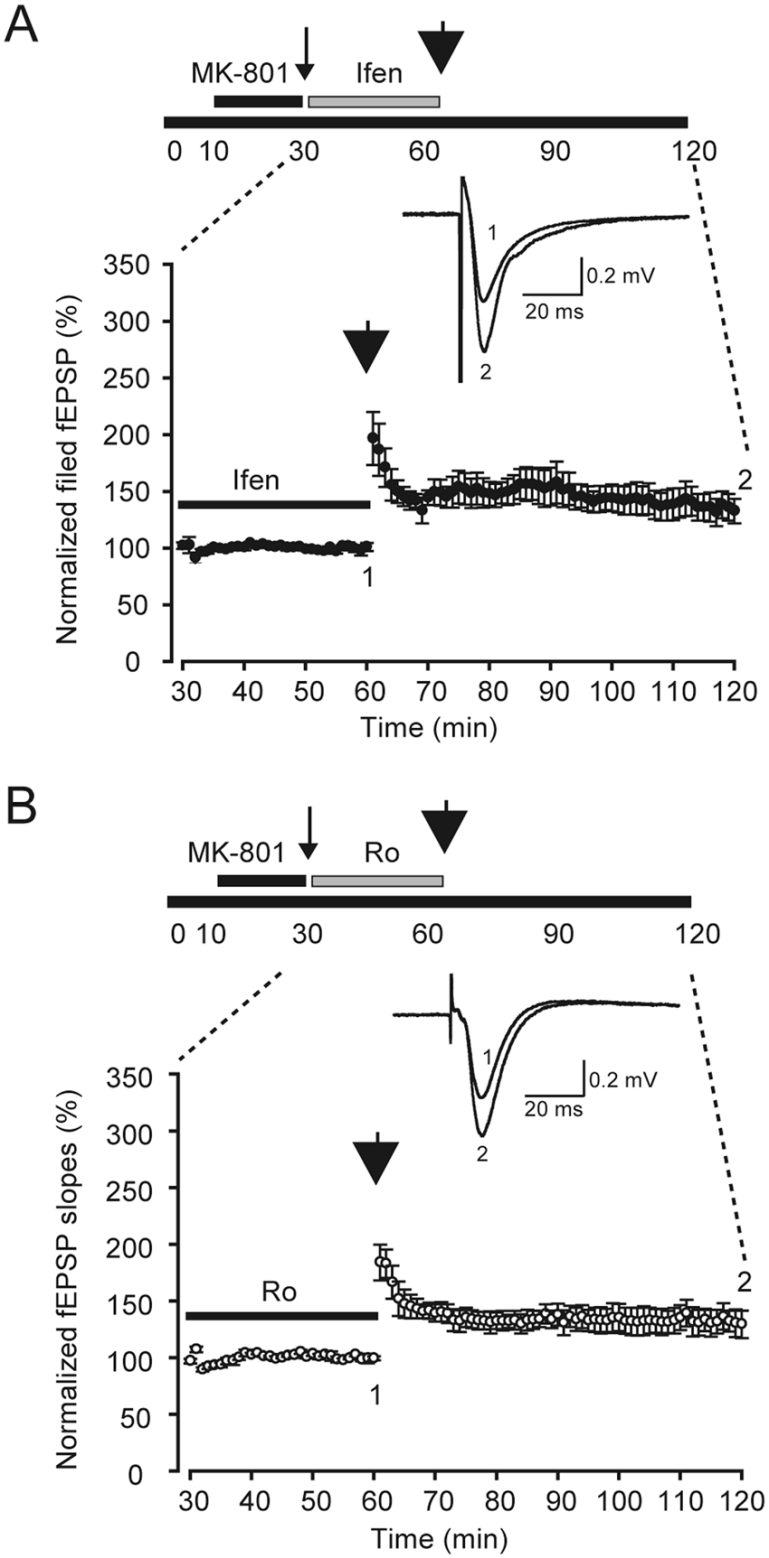
LTP when S-NMDARs were inhibited. LTP was induced in the presence of ifenprodil (**A**, closed circles, n = 6) or Ro (**B**, open circles, n = 5). LFS and 3-train HFS are represented by thin and thick arrow heads, respectively. Ifenprodil (Ifen, 10 μM) or Ro25-6981 (Ro, 3 μM) application is indicated by the horizontal black bar. Insets: traces recorded at various time points.

To test the LTP of CA1 neurons, the fEPSPs were measured through extracellular field potential recordings. We unexpectedly observed that the slope of fEPSPs (Fig. 2A, 137.2% ± 12.2%, n = 6) had increased and lasted longer than 1 h after the selective blocking of S-NMDARs and treatment with ifenprodil (a selective NR2B antagonist). This indicates that robust LTP is evoked by 3-train HFS in such situations. To confirm whether the findings occurred because of the effect of ifenprodil or the blocking of extrasynaptic NR2B-containing NMDARs, Ro (another selective NR2B antagonist) was used. Similar results were obtained; the slope of fEPSPs (Fig. 2B, 132.6% ± 11.0%, n = 5; *p* = 0.546, compared with ifenprodil results) had increased and lasted longer than 1 h. Thus, LTP can be induced when S-NMDARs and NR2B-containing ES-NMDARs are blocked. Because this LTP is induced after blocking S-NMDARs, we named it the ex-LTP in this paper.

### LTP cannot be induced when S-NMDARs are selectively blocked

**Figure 3:**
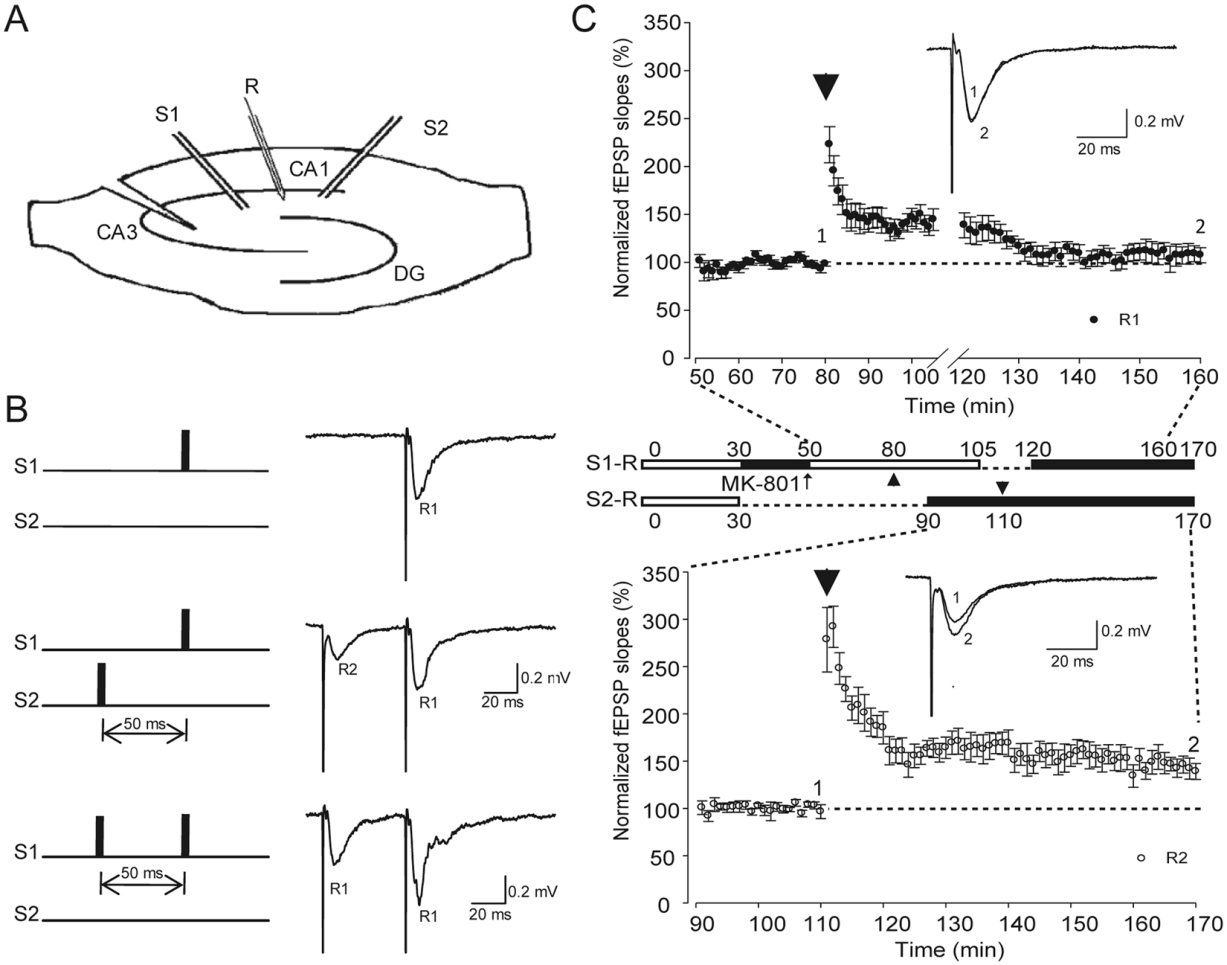
Comparison of LTP induction in two independent pathways. (**A**) Schematic diagram showing the two-pathway experiment. (**B**) Paired pulse facilitation tests of the two pathways. *Upper:* Stimulation of S1-evoked field EPSP (R1); *Middle*: Stimulation of S2 (50 ms prior to the stimulation of S1), which did not change in the amplitude of R1; *Lower*: Paired pulse in the same inter-stimulus interval in S1 facilitated R1. (**C**) LTP inductions in two pathways of the same slices. Single test stimuli were delivered to S1 and S2 alternatively every 30 s. *Upper*: Summary of normalized fEPSP slopes in S1-R pathway (n = 7). *Middle*: MK-801application (30–50 min) followed by 5 Hz stimulation in S1 (16 s). From 105 to 120 min, single test stimuli were stopped upon the delivery of HFS to S2, to avoid heterosynaptic plasticity. *Lower*: Summary of normalized fEPSP slopes in the S2-R pathway (n = 7). LFS and 3-train HFS are represented with thin and thick arrow heads, respectively.

To test whether the same HFS can induce LTP when S-NMDARs were blocked or not, we designed a two-pathway experiment, as shown in Fig. 3. Two independent pathways (S1-R and S2-R) were stimulated in the same slice, and their independency was confirmed through the paired pulse facilitation test. The stimulation of S2, which occurred 50 ms prior to the stimulation of S1, did not alter the amplitude of S1-evoked fEPSPs (Fig. 3B, middle trace). By contrast, the paired pulse of S1 at the same inter-stimulus interval evoked the facilitation of R1 (Fig. 3B lower trace). After MK-801 had been applied to the slice for 20 min, the S1-R pathway was stimulated by 5-Hz stimulation for 16 s to inhibit the S-NMDARs. The S2-R pathway did not receive stimulation, and these S-NMARs were therefore expected to be unrestrained. We then compared the LTP induction between the two pathways. In the S1-R pathway, HFS applied to S1 failed to induce LTP (Fig. 3C, upper, closed circles; 109.7% ± 8.2%, n = 7). By contrast, HFS applied to S2 did induce a robust LTP in the S2-R pathway (Fig. 3C, lower, open circles; 145.9% ± 9.3%, n = 7; *p* = 0.013, compared with the S1-R pathway). It confirmed that MK-801 did not damage the slices and that the S-NMDARs were not blocked without the 5-Hz stimulation. These results suggested that MK-801 with 5-Hz stimulation can selectively block S-NMDARs, and LTP cannot be induced by the same HFS when S-NMDARs were blocked.

### Extrasynaptic NR2A-containing NMDARs are necessary for ex-LTP induction

**Figure 4:**
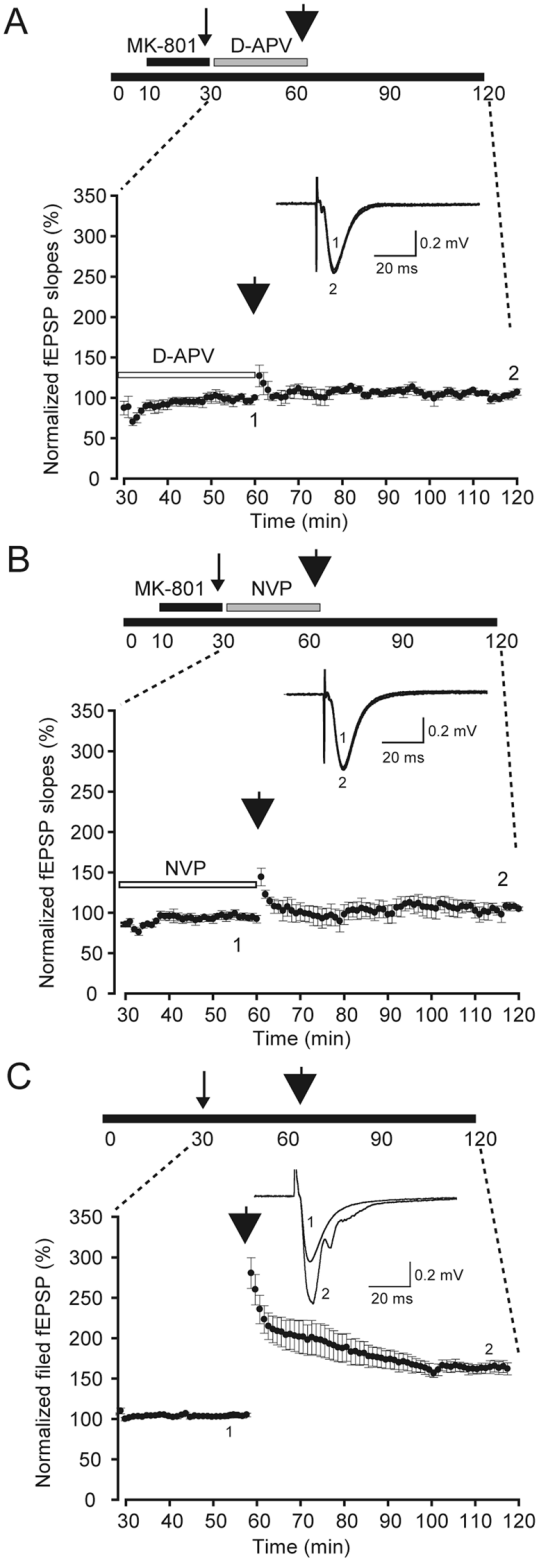
LTP induction in different situations. (**A**) Lack of LTP induction after obstruction of both S-NMDARs and ES-NMDARs (n = 4). D-APV was applied for 30 min after MK-801 was washed out. (**B**) Lack of LTP induction when the extrasynaptic NR2A subunit was blocked (n = 6). NVP-AAM077 (0.1 μM) was applied for 30 min after MK-801 was washed out. (**C**) LTP induction after 5-Hz stimulation for 16 s (n = 11). *Upper*: Schematic diagram of the experiment protocol. *Lower*: The summary of normalized fEPSP slopes from the 30^th^ min to the 120^th^ min is provided. *Inset*: sample traces of EPSC at timings 1 and 2, as marked. Thin arrow heads indicate the time when the 16 s 5-Hz stimulation was delivered; thick arrow heads indicate the time when the three trains of HFS were delivered.

To determine whether the ex-LTP is depended on extrasynaptic non-NMDARs, we blocked the S- and ES-NMDARs by using 100 μM D-AP5 and then delivered 3-train HFS into the slices. LTP was not observed in any of the D-APV applied slices (104.8% ± 1.5%, n = 4, Fig. 4A), which indicated that ex-LTP was impaired after the blockade of both S-NMDARs and ES-NMDARs. It suggests that the ex-LTP is ES-NMDAR dependent.

NMDARs are primarily heteromeric assemblies of NR1, NR2 and NR3. The NR2 subunit determines many of the properties and functions of NMDARs. In adult hippocampus, NR2A and NR2B are the predominant NR2 subunits. Here, the ex-LTP was induced when extrasynaptic NR2B-containing NMDARs were blocked (as shown in Fig. 2). To examine whether blocking only NR2A-containing NMDARs could also induce LTP, we applied NVP; subsequently 3-train HFS was delivered into the slices. No LTP was observed (103.6% ± 5.0%, n = 6, Fig. 4B), indicating that LTP could not be induced when extrasynaptic NR2A-containing NMDARs were inhibited.

### Comparison of the size of different LTP

**Figure 5:**
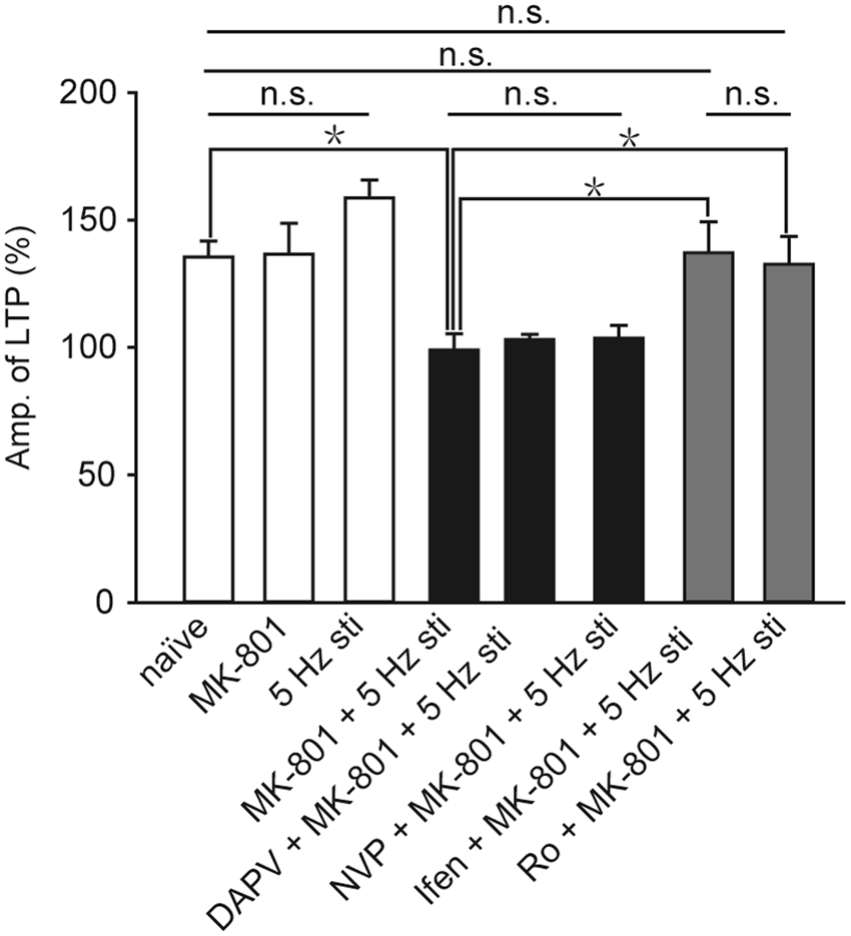
Comparison of LTP values among different experimental groups. Naïve, n = 4; MK-801, n = 6; 5 Hz stimulation, n = 11; MK-801 and 5 Hz stimulation, n = 9; DAPV, MK-801 and 5 Hz stimulation, n = 4; NVP, MK-801 and 5 Hz stimulation, n = 6; Ifen, MK-801 and 5 Hz stimulation, n = 6; Ro, MK-801 and 5 Hz stimulation, n = 5. All data points represented as mean ± SE, *p < 0.05.

To test whether this 5-Hz stimulation affects induction of LTP or not, 3-train HFS was delivered 30 min after the 5-Hz stimulation. As shown in Fig. 4C, applying 3-train HFS could induce LTP even after the 5 Hz priming stimulation, and the amplitude was not significantly different from the one induced by the same HFS in naïve slices (*p* = 0.088, Fig. 5).

**Figure 6:**
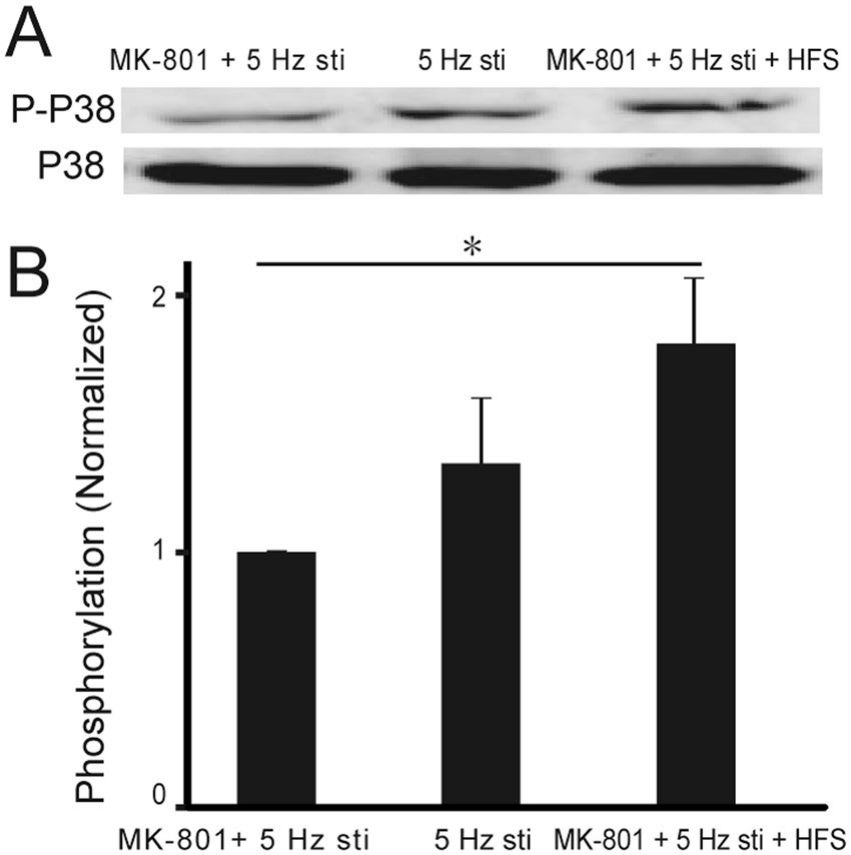
HFS induced phosphorylation of p38 MAPK when S-NMDARs are blocked. (**A**) Western blots of the indicated phosphoproteins in CA1 neurons. (**B**) The summary histograms quantifying relative immunoreactivity. Blots are cropped for clarity; full blots are shown in the Supplementary file. All data points represented as means ± SE, n = 3, *p < 0.05.

To compare the LTP values in different conditions, a summary is depicted in Fig. 5. When the slices were treated with only MK-801, LTP of the CA1 neurons could be induced, and these LTP values were not significantly different from the naïve control group (*p* = 0.947) as shown in Fig. 6 blank bars. By contrast, when the slices were treated both with MK-801 and 5-Hz stimulation (99.0% ± 6.4%, n = 9), LTP of CA1 neurons cannot be induced, and the LTP values were not changed by the application of D-APV (*p* = 0.587, compared with MK-801 and 5-Hz stimulation) or NVP (*p* = 0.676, compared with MK-801 and 5-Hz stimulation) as shown in Fig. 6 black bars. Moreover, when the slices were treated both with MK-801 and 5-Hz stimulation, LTP of neurons in CA1 were induced after adding selective NR2B antagonist (ifenprodil or Ro) as shown in Fig. 6 (gray bar); these LTP values were not significantly different from those normal LTP which induced in naïve hippocampal slices (Ifen + MK-801 + 5 Hz, *p* = 0.919; Ro + MK-801 + 5 Hz, *p* = 0.498). In short, in all of our experiments where S-NMDARs were blocked, LTP was induced by HFS only when the NR2B-containing NMDARs were blocked. These results implicate the blockade of S-NMDARs can impair LTP induction, which can be restored by the application of NR2B antagonist.

### Activation of ex-NMDARs enhanced phosphorylation of p38 MAPK

MAPK signaling pathway is reported to be involved in neural plasticity. And p38 MAP kinase activity has been reported to causes the downregulation of CREB protein and may lead to impair of LTP induction. In addition, previous work has reported that NR2B antagonist reduces the activation of p38 MAPK. Thus we performed western blotting experiments to test whether phosphorylation of p38 MAPK was changed with the activation of ES-NMDARs. As shown in Fig. 6, the phosphorylation level of p38 MAPK was significantly increased by the activation of ES-NMDARs (*p* = 0.04, compared to MK-801 + 5 Hz group), indicating that the activation of ES-NMDARs increases the phosphorylation of p38 MAP, which may increase the induction threshold of ES-NMDARs dependent LTP.

## Discussion

In this study, we used MK-801 to block S-NMDARs and investigate the role of ES-NMDARs in the LTP of mature mouse hippocampus slices. Voltage-clamp recordings demonstrated that the single test stimuli could not induce any NMDA-EPSCs in 30 min after the washout of MK-801, indicating that most of the active S-NMDARs in the same input pathway were irreversibly blocked (Fig. 1). Extracellular field potential recordings revealed that no LTP could be induced by HFS (Fig. 3), but that after the blocking of extrasynaptic NR2B-containing NMDARs, LTP was restored (Fig. 2). Furthermore, this extrasynaptic LTP was supposed to be ES-NMDARs dependent, because LTP could not be induced if all of the NMDARs were blocked (Figs 4 and 5). To the best of our knowledge, this is the first report on ES-NMDAR-dependent LTP.

We used a 5-Hz stimulation to selectively open S-NMDARs and applied MK-801 to selectively block S-NMDARs. Because of the diverse locations of S- and ES-NMDARs, the kinetics of the induced currents (EPSCs) were different when glutamate combined with S- and ES-NMDARs^22^. Variations in the rising time and decay time constant of NMDA-EPSCs were used to distinguish the currents from S- and ES-NMDARs. Under our experimental conditions, the rising time and decay time constant of NMDA-EPSCs did not statistically vary with the 5-Hz stimulation, indicating that extrasynaptic NMDA-EPSCs were not activated by the stimuli, as previously reported^19^.

For the trafficking of NMDARs, earlier experiments using cultured hippocampal neurons showed that ES-NMDARs are highly mobile at the surface of neurons and able to diffuse into the synaptic compartment within minutes^23, 24^. This raises the question of whether the ES-NMDARs diffuse into synaptic area, leading to incomplete inhibition of S-NMDARs before HFS is applied. It has been reported that the lateral mobility of NMDARs is less than α-3-hydroxy-5-methylisoxazole-4-propionie-acid receptors (AMPA receptors), and changes in neuronal activity do not modify NMDAR mobility^24, 25^. In addition, Harris and Pettit^6^ reported that the pool of ES-NMDARs is stable and does not shuttle into the synaptic receptor pool in the hippocampal slices. They proposed that ES-NMDARs in the intact tissue may be stabilized by extracellular matrix cues. We observed here no recovery of S-NMDA-EPSCs within at least 30 min after washout of MK-801 (Fig. 1), indicating that the blockade of S-NMDARs was stable as fast as in 30 min under our experimental conditions. Taken together, in our experimental conditions, the ex-LTP should be evoked, at least mainly, via the activation of ES-NMDARs.

In previous work, it suggested that 5-Hz stimulation can inhibit the LTP induced by one train HFS, and NR2B-containing NMDARs was required for this metaplasiticity^19^. The HFS used here is 3-train, which is stronger than one train. It suggested that the priming stimulation increases the threshold of LTP but not block plasticity, which is accordance with previous report^26^. The restore of ex-LTP by blockade of NR2B-containing ex-NMDARs suggests that activation of extrasynaptic NR2B plays a role in this type of metaplasticity.

The NR2 subunit composition of NMDARs varies during development. During early postnatal development, the cellular and synaptic expression of NR2A increased from near background levels at P2 to adult levels by P15; the cellular NR2B expression was at adult level by P2, and synaptic NR2B decreased with age^27^. In the adult hippocampus, NR2A is enriched in synapses and NR2B is primarily in extrasynaptic sites^5^. So far, many studies have demonstrated the involvement of NMDARs and the important (positive or negative) roles of individual NR2 subunits in LTP induction: Some reports suggest that NMDARs containing NR2A or NR2B subunits promote LTP while others against them^10–13^. Few of them, however, were focused on the role of synaptic- or extrasynaptic locations of NR2A/2B, since the 100 Hz stimulation, which is commonly used to induce LTP, evokes both S-NMDARs and ES-NMDARs^21^. Our studies have shown that LTP induction is restored in ES-NMDAR (Fig. 2) procedures in the presence of NR2B antagonist, thereby first provide evidences that the extrasynaptic NR2B-containing NMDARs modulate induction threshold of LTP.

MAPK signaling pathway is supposed to be involved in neural plasticity. p38 MAP kinase activity has been most often associated with LTD, and can cause the down-regulation of CREB protein and may lead to impair of LTP induction. Our results of western blotting experiments indicate that when S-NMDARs are blocked, HFS will increase the phosphorylation level of p38 MAPK. It has been reported that NR2B inhibitors can decrease the phosphorylation level of p38 MAPK^28^. In Alzheimer’s disease, accumulation of Aβ can mediate rise in p38 MAPK activation. In HD mouse striatum, specifically increased extrasynaptic NMDAR current and associated reductions in nuclear CREB activation were reported, and pharmacological block of extrasynaptic NMDARs with memantine reversed signaling and motor learning deficits^18^. Metaplasticity which can alter the threshold for LTP induction may support as a way to compensate a situation that the S-NMDARs lost their functions, and learning and memory was impaired.

In the present study, we succeeded in selectively activating the ES-NMDARs and separately investigating the roles of the NR2A and NR2B subunits at the extrasynaptic site in adult mice hippocampal slices. An ex-LTP was induced when synaptic and extrasynaptic NR2B-containing NMDARs were blocked. This ex-LTP was demonstrated to be extrasynaptic NMDARs dependent, and it is kind of metaplasticity which modulated by NR2B. Moreover, whether NR2B antagonists can impair learning and memory deficits in Alzheimer and Huntington’s diseases can be further studied.

## Materials and Methods

### Slice preparations

Hippocampal slices were prepared as described previously^19^. C57BL/6 J mice (male; 7–8 weeks of age) were deeply anesthetized with halothane, and the hippocampi were then rapidly separated from the brain following decapitation. Transverse hippocampal slices (400 µm thick) were cut at 0–4 °C with a vibratome tissue slicer and were placed in a humidified interface-type chamber at 25–26 °C for at least 2 h in the presence of a gas- saturated (95% O_2_, 5% CO_2_) artificial cerebrospinal fluid (ACSF) containing the following (in mM): 119 NaCl, 2.5 KCl, 2.5 CaCl_2_, 1.3 MgSO_4_, 1.0 NaH_2_PO_4_, 26.2 NaHCO_3_, and 11 glucose. All experiments were performed in accordance with the guidelines of the Institutional Animal Care and Use Committee (IACUC) of Shanghai Jiao Tong University, and all experimental protocols were approved by the IACUC, Shanghai, China.

### Electrophysiology

Individual slices were transferred into a submerge-type recording chamber, fixed with a nylon net, and submerged beneath the continuously perfused gas-saturated ACSF (at a rate of 2.0 mL/min). In all of the experiments, a γ-aminobutyric acid receptor (GABA_A_) antagonist picrotoxin (100 µM) was included in the ACSF. A cut was made to separate the CA3 region from the CA1 region to avoid epileptiform activity from the CA3 region. All experiments were performed at 25 °C by using a temperature controller (HCC-100A, Dagan Corporation, Minneapolis, MN).

The Schaffer collateral-commissural fibers in the stratum radiatum of the CA1 region were stimulated every 15 s through a bipolar tungsten stimulating electrode with 0.2-ms constant-current pulses. Whole-cell patch-clamp recordings were obtained from CA1 pyramidal cell bodies using an IR-CCD camera-installed (Hamamatsu Corporation, Shizuoka, Japan) upright microscope (Olympus BX50WI, Tokyo, Japan). The patch electrodes (4–6 MΩ resistance, Harvard Apparatus, Holliston, MA) were filled with an internal solution containing the following (in mM): 122.5 Cs gluconate, 17.5 CsCl, 10 HEPES, 0.2 EGTA, 8 NaCl, 2 MgATP, and 0.3 Na_3_GTP (pH 7.2, 290–310 mOsm). The values of the membrane potential were compensated for the liquid junction potential at the electrode tip. Series and input resistances were monitored throughout each experiment. The cells were excluded from data analysis if a >25% change in series resistance occurred during the experiments. NMDAR-mediated excitatory postsynaptic currents (NMDA-EPSCs) were recorded in the voltage-clamp mode at +40 mV in the presence of 10 µM 6-cyano-7-nitroquinoxaline-2,3-dione (CNQX), a non-NMDAR antagonist throughout the whole-cell patch clamp experiment. The strength of single test stimuli was adjusted to maintain the NMDA-EPSC amplitudes within a range of 60–150 pA.

Extracellular field potentials were recorded in the stratum radiatum by using a glass recording pipette filled with 3 M NaCl (3–6 MΩ resistance). The amplitudes of the field excitatory postsynaptic potentials (fEPSPs) were calculated as the initial slope of the EPSP. The stimulus strength was adjusted to 6–25 pA so that EPSPs of 0.15–0.20 mV/ms initial slopes were evoked.

In both whole-cell patch clamp recording and field potential recording, S-NMDARs were blocked by delivering a short train of low-frequency stimulation (LFS, 5 Hz for 16 s) to slices pretreated with irreversible NMDAR antagonist MK-801 for 20 min. To induce sustained LTP in the field potential recordings, three trains (at 15 s intervals) of high-frequency stimulation (HFS, 100 Hz/1 s) were applied after washing out MK-801. The LTP values were calculated as the ratio of the average stable response after induction (at 55–60 min after HFS) and before the induction of LTP (at 0–30 min before HFS). Usually, LTP is successfully induced if the LTP value is >120%. Both LFS and HFS were delivered at identical intensity as the basal test stimulation.

‘Two pathway’ experiments were performed by placing an additional stimulation electrode in the CA1 area of the Schaffer collaterals (on the border of the subiculum). The stimulation intensity of S1 was adjusted to 0.15–0.2 mV/ms initial field EPSPs, and the stimulation intensity of S2 was to evoke 0.045–0.075 mV/ms field EPSPs (30–50% of R1). But identical intensity was used for paired pulse, test and induction stimuli in the same pathway. The two stimulation electrodes (S1 and S2) were activated alternately once every 15 s with stimuli separated by 30 s in the same pathway in order to stimulate two separate synaptic populations. Crosstalk between synaptic populations was minimized by assuring that there was neither paired-pulse facilitation nor paired-pulse depression when stimuli were separated by 50 ms. During delivering HFS/LFS in one pathway, basic stimulation in another pathway was paused ahead of 5 min lasting 15 min.

A Multiclamp 700B amplifier (Molecular Devices, Union City, CA) was used in all experiments, and the records were kept in a personal computer and analyzed (filtered at 3 kHz, sampled at 10 kHz) using PClamp 10 (Molecular Devices, Union City, CA).

### Western blotting

For one western blotting experiment, 10–15 pieces of CA1 tissues were collected for every situation. Samples were homogenized and total proteins were extracted using RIPA lysis buffer (Sigma, USA), including protease and phosphorylase inhibitors (Roche, USA). The blots were incubated overnight at 4 °C with rabbit polyclonal antibodies: p-p38 and p38 (1:1000; Cell Signaling, USA), and mouse monoclonal anti-GAPDH antibody (1:5000; Beyotime, China). Membranes were rinsed and incubated for 1 h with fluorescence-conjugated goat anti-rabbit (1:5000; Thermo Scientific, USA) and anti-mouse (1:5000; Millipore, USA). Blots were scanned using an Odyssey system. p-p38 and p38 levels were quantified through densitometry using ImageJ software.

### Chemicals

CNQX, MK-801, ifenprodil, and Ro25-6981 were obtained from Tocris Cookson (Bristol, UK), and picrotoxin was obtained from Sigma-Aldrich (St. Louis, MO). NVP-AAM077 (NVP) was obtained from Professor Yao’s lab at the Shanghai Institute of Organic Chemistry, Chinese Academy of Sciences.

### Statistical analysis

All data are expressed as mean ± SEM. Within-group comparisons were performed using unpaired Student’s two tailed t-tests, and the differences between groups were compared using ANOVA post hoc comparisons. An ANOVA post hoc least significant difference test was used when equal variances were assumed. Statistically significance was set at *p* < 0.05.

## Electronic supplementary material

full-length blot of figure 6
