# Supplementary material for: Extrasynaptic NMDA receptor dependent long-term potentiation of hippocampal CA1 pyramidal neurons
Source: Sci Rep. 2017 Jun 8;7:3045. doi: 10.1038/s41598-017-03287-7 (PMC5465207; doi:10.1038/s41598-017-03287-7)
Supplement: Supplementary file 1 — full-length blot of figure 6 [file 41598_2017_3287_MOESM1_ESM.pdf]

Extrasynaptic NMDA receptor dependent long-term potentiation of hippocampal CA1 pyramidal neurons

Qian Yang[1#], Geng Zhu[1#], Dandan Liu[1], Jue-Gang Ju[1], Zhen-Hua Liao[1], Yi-Xin Xiao[1], Yue Zhang[1], Naijian Chao[1], JieJie Wang[2], Weidong Li[1], Jian-Hong Luo[2*], Sheng-Tian Li[1*]

[1]Key laboratory for the Genetics of Developmental and Neuropsychiatric Disorders (Ministry of Education), Bio-X Institutes, Shanghai Key Laboratory of Psychotic Disorders, Institute of Social Cognitive and Behavioral Sciences, and Brain Science and Technology Research Center, Shanghai Jiao Tong University, Shanghai, China.

[2]Department of Neurobiology, Key Laboratory of Medical, Neurobiology (Ministry of Health of China), Collaborative Innovation Center for Brain Science, School of Medicine, Zhejiang University, Hangzhou, Zhejiang 310058, China

[*] luojianhong@zju.edu.cn; Tel.: +86-571-88208002; Fax: +86-571-88208002

[*] lstian@sjtu.edu.cn; Tel.: +86-21-34208036; Fax: +86-21-34208035

[#] contributed equally to this work.

Figure 6

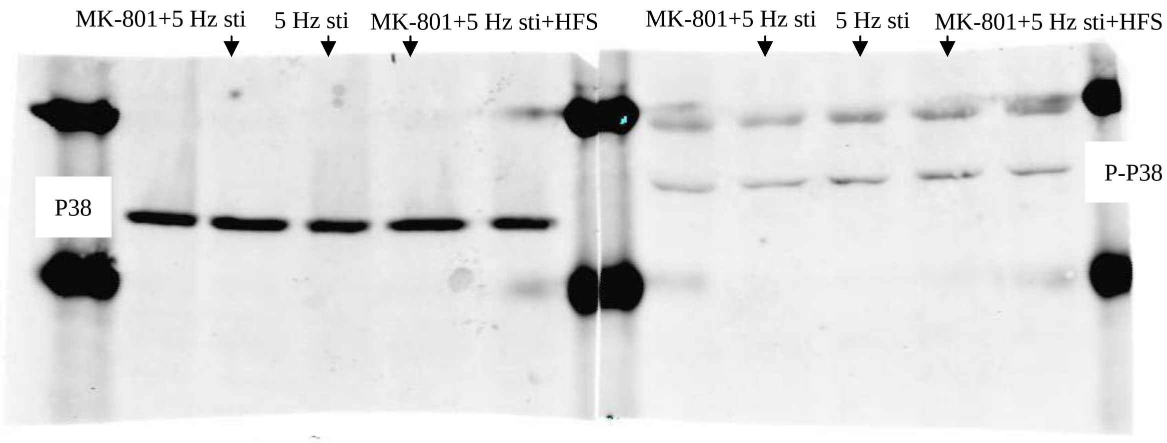

Figure S1. Full-length blots for the indicated figures from the main text.
